# Child health promotion during the COVID-19 pandemic: A health and welfare sector collaboration

**DOI:** 10.4102/phcfm.v15i1.3767

**Published:** 2023-02-13

**Authors:** Nompumelelo Ntshingila, Alida S. du Plessis-Faurie

**Affiliations:** 1 Department of Nursing, Faculty of Health Sciences, University of Johannesburg, Johannesburg, South Africa

**Keywords:** children, collaboration, COVID-19 pandemic, health promotion, well-being

## Abstract

**Background:**

Fragmented service provision and a lack of efficient cooperation between health and welfare sectors serving children and families remain ongoing challenges in South Africa. The coronavirus disease 2019 (COVID-19) pandemic escalated this fragmentation. A community of practice (CoP) was established by the Centre for Social Development in Africa to promote collaboration between the sectors and to assist communities in their environments.

**Aim:**

To explore and describe collaboration on child health promotion between professional nurses and social workers, who formed part of the CoP during the COVID-19 pandemic.

**Setting:**

The study was conducted in five public schools from four of the seven district regions of the City of Johannesburg, Gauteng province.

**Methods:**

A qualitative, exploratory, descriptive research design was employed to conduct psychosocial and health screenings of children and their families. Focus group interviews were conducted, and field notes were used to collect and confirm data from the team.

**Results:**

Four themes emerged. Participants shared their positive and negative experiences faced during the fieldwork, their realisation of the value of collaboration between various sectors and their desire and capacity to do more.

**Conclusion:**

Participants indicated that collaboration between the health and welfare sectors is vital to support and promote the health of children and their families. The COVID-19 pandemic highlighted the need for collaboration between these sectors in the children and their families’ ongoing struggles.

**Contribution:**

The importance of these sectors being engaged as a team highlighted the multisectoral influence shaping child development outcomes, supporting children’s human rights and advancing social and economic justice.

## Introduction

This article focuses on exploring and describing the experiences of a community of practice (CoP) of professional nurses collaborating with social workers, who conducted psychosocial and health screening of children and their families in Gauteng. Even though the Integrated School Health Policy (ISHP), the World Health Organization (WHO), the United Nations International Children’s Emergency Fund (UNICEF) and the World Bank encourage multisectoral collaboration, research indicates collaboration between various sectors is insufficient. The inclusive healthcare needs of children and their families are not met, and service provision is fragmented. Practical cooperation between health and welfare sectors serving children and families was confirmed as being challenging.^1^

The South African government has undertaken to ‘put children first’ by becoming a signatory to the United Nations Convention on the Rights of the Child^2^ and by distinctly recognising children in the Bill of Rights of the South African Constitution.^3^ This pledge aims to guarantee that children’s rights are upheld and delivered to support all children in reaching their full potential. This undertaking is particularly important in the foundational school phase where, if special attention is given to children’s optimal health, their immediate survival, growth and health are likely to be enhanced. Their long-term learning outcomes and development are also promoted.^4^

However, assuring all children’s optimal development presents a significant challenge,^4^ particularly during the coronavirus disease 2019 (COVID-19) pandemic. Research undertaken by the Human Rights Watch^5^ confirmed that the pandemic intensified previously existing disparities, and children who were already at risk of being left out from quality education are among the most affected. Moreover, South Africa’s high rate of income poverty among children is a known risk factor that negatively influences child well-being in different domains, including health, nutrition, education, safety and security and psychosocial health. More than six out of 10 children (62.1%) are acknowledged to experience multifaceted poverty,^6^ implying that their most basic needs for nutrition, clothing and shelter cannot be met.

Therefore, meeting the multidimensional needs of children in different stages of their growth and development remains a significant national priority, as outlined by the National Planning Commission.^7^ This priority is central to the achievement of children’s rights to basic education, health, food, care and social assistance, as guaranteed in Sections 27, 28 and 29 of the Constitution of the Republic of South Africa of 1996.^3^ In addition to advancing children’s rights, investments in children’s nutrition and health have short-term and long-term effects.^8,9^

To address children’s immediate health problems, the ISHP was developed in 2012.^4^ The ISHP intended intersectoral collaboration between the Department of Health (DoH), the Department of Basic Education (DoBE) and the Department of Social Development in order to contribute to the development of sustainable and comprehensive school health programmes.^1^ Strengthened school health services represent one of the key components of the health sector’s efforts to re-engineer and promote primary health care delivery.^4^ The National Health Insurance Policy for schools states that health services should form part of the re-engineered primary health care services stipulated by the DoH.^10^ Additionally, the ISHP emphasises the importance of integrating local health agencies, such as primary health care facilities, into school health services.^4^ The WHO, UNICEF and the World Bank’s framework^11^ propose that nurturing environments should be created to integrate various interventions across different social sectors and promote better outcomes for children in the early years.

Primary health care delivery, coupled with promoting child well-being outcomes through multifaceted domains, are important social investments in human capital development.^12^ The policy could hold long-term benefits for children as individuals and have a positive effect on their families by promoting economic development. Improved education outcomes enhance employability and better income in adulthood.^13^ Further benefits include social cohesion and political stability.^12,14^

In 2019, Rasesemola et al.^1^ conducted a survey in Gauteng to determine the province’s level of compliance with the ISHP. The research findings confirmed that intersectoral and multidisciplinary collaboration between schools and other stakeholders to ensure compliance with the ISHP was nonexistent. Moreover, school health nurses’ roles were ambiguous^15^; these nurses are primarily contracted to the DoH, and their nonpermanent affiliation with the DoBE (being the steward of schools) means their placement or visits to schools are intermittent. Therefore, they provide services as guided by the ISHP, as they are not appointed as full-time school-based healthcare professionals employed by the DoBE.

School social work is also a reasonably new development in the current South African education system.^16^ Consequently, the roles and responsibilities of school social workers are unclear to their employers and the larger education system, leading to disarray, not only for the DoBE, the school governing bodies and school principals but for the social workers themselves.^16^ The reason for this situation is that social work, as a profession, is mandated by a code of professional ethics and conduct and specific legislative requirements set out in the Constitution of South Africa, the *Social Service Professions Act* and the *Children’s Act*. Also, school social workers are duty-bound by the expectations and ethical codes of the South African Council for Social Service Professions.^17^

To remedy the mentioned ambiguity, an intersectoral CoP was established in 2020 by a multidisciplinary team of researchers and practitioners from the University of Johannesburg and the University of the Witwatersrand, Johannesburg, to search for innovative outcomes suited to the South African context. Key stakeholders from health, welfare, social work, education, mental health and a team of engineers collaborated in the development and testing of a digital tool to assess and track child well-being and to devise intervention plans for children and families with different risk profiles. The Child Well-being Tracking Tool (CWTT) was implemented with 181 children aged 6–8 years old (Grade R and Grade 1) in five schools in the poorest wards of the City of Johannesburg at different stages of the national lockdown because of the COVID-19 pandemic. Children in the foundational years of schooling were selected, as their early learning is remarkably significant because it strongly predicts later success in thinking and development.^18^ Literature further confirms that a direct correlation exists between a child’s self-esteem and ability to learn with ease at school.^19^ Being healthy and living in favourable circumstances boosts learning, which is considered a valuable and effective way of investing in the future.^20^ A study undertaken by Claudete and Claire^21^ found a statistically noteworthy link between foundation year learning and the socio-economic transformation of communities.

Research undertaken by Mabasa^22^ reiterates that an approach involving multiple role-players, skills capacity and knowledge is the definitive way forward, re-addressing society, culture and socio-economic arrangements. In this study, health and welfare sectors’ role-players focused on strengthening and promoting functional collaboration to address the needs of vulnerable learners. Both sectors agreed that collaboration between the health and welfare sectors was vital to support and promote the health of children and their families. This pilot study confirmed that amidst the COVID-19 pandemic, parents living in deprived circumstances were unable to support their children holistically. It was thus determined that multisectoral influence from both sectors is required in shaping child development outcomes, supporting children’s human rights and advancing social and economic justice. The aim of this study was to explore and describe collaboration on child health promotion between professional nurses and social workers, who formed part of the CoP during the COVID-19 pandemic.

## Research methods and design

### Study design

A qualitative, exploratory, descriptive research design, as described by Holloway and Gavin^23^ and Ravitch and Carl,^24^ was used in this study. A qualitative research design was considered suitable for this study because the researcher wanted to explore and understand the experiences of professional nurses and social workers conducting psychosocial and health screenings with Grade R children, Grade 1 children and their families. An exploratory research design was employed because little knowledge about intersectoral collaboration in South Africa was available. The nature of professional nurses’ and social workers’ experiences during this research was investigated. It was envisaged that a descriptive research design would best portray the experiences of the professional nurses and social workers in conducting psychosocial and health screenings with Grade R children, Grade 1 children and their families in the various regions.

### Setting

The CoP researcher recruited professional nurses and social workers who were competent to conduct psychosocial assessments and health screenings in the homes of the Grade R children, Grade 1 children and their families, based in four regions in Gauteng. Region A includes the Diepsloot-to-Midrand belt; Region C includes Roodepoort and surrounding areas; Region E consists of Alexandra, Wynberg and Orange Grove, extending as far as Region F,^25^ which includes Johannesburg inner city and Johannesburg south.^26^

The Grade R and Grade 1 children were identified from their schools and followed up with at their homes. The home settings where the psychosocial and health screenings were conducted ranged from informal to formal settlements. The assessments and screenings were conducted at the homes of the children because, at that time, the COVID-19 pandemic was at its peak. The team of professional nurses and social workers carried out the fieldwork from September 2020 until November 2020. At the end of November 2020 (post-data collection), the CoP researcher conducted focus group interviews with the social workers and professional nurses in a boardroom at a university in Johannesburg.

### Target population and sample

The study’s target population consisted of social workers and professional nurses. From this target population, a sample of seven social workers and six professional nurses were recruited to implement psychosocial and health screenings. The participants’ ages ranged between 22 and 50 years. Participants’ experience in their professional capacity was 3 years and longer. They held bachelor’s degrees in nursing and in social work.

### Sampling strategy

Purposive sampling was used in this study. Purposive sampling involves researchers’ conscious selection of participants.^27,28^ In this study, the professional nurses and social workers were identified by the researcher because they had relevant, first-hand experience and information related to the CoP. The participants conducted the psychosocial and health screenings with Grade R children, Grade 1 children and their families.

### Data collection

Data were collected using focus group discussions and field notes. During the focus group, members had to reflect on similar matters to construct meaning of their experiences, as proposed by Nyumba, Wilson, Derrick and Mukherjee.^29^ Data were collected in November 2020. Open-ended interview questions were posed to the participants based on the interview guide, as indicated in Box 1. Ultimately, the researcher transcribed the focus group discussions.

BOX 1 Interview guide for the focus groups.

**Interview guide**

- What were your experiences, thoughts and observations when working with the children?
- What were your experiences, thoughts and observations when working with the parents?
- What stood out for you during the data collection period?
- What worked to ensure better data collection with the children? Or what assisted you to engage better with the children?
- Looking back now, what could you have done differently?

### Data analysis

The interview guide (Box 1) directed the researchers’ analysis; thematic data analysis^30^ was thus carried out by the research team. The researcher carefully perused all the transcripts and identified concurrent themes independently. All transcripts were read and reread to ascertain the underlying meaning. The researcher compiled a list of all the themes and grouped similar topics together. Columns were drawn, and data were sorted according to major categories, subcategories and emerging themes. The list of themes was abbreviated into codes and placed next to the appropriate segments of text. This procedure was followed to ensure all categories and codes were covered. Themes relating to one another were grouped together, and the researcher associated categories by drawing lines between similar categories to indicate interrelationships. Subsequently, all the material was assembled into one document, and categories were coded where necessary. All the collected data were backed up to ensure security.

### Trustworthiness

Measures to ensure trustworthiness were maintained, as stipulated by Lincoln and Guba.^31^ Credibility was ensured by triangulating data collection using focus group discussions and field notes. The research method employed in this study has been described in detail to ensure the study can be replicated. Moreover, research findings have been supported by recent literature sources to enhance credibility. The researcher also built strong and trusting relationships with the participants, which enhanced the credibility of the findings shared during the data collection process.

Participants were selected using purposive sampling, and a rich description of the findings supported with direct quotes was used to ensure transferability. Dependability was promoted by the researcher keeping personal and field notes during the data collection process. Confirmability was also achieved through the research leaders’ presence during the data collection process to audit the research project. An independent coder analysed the collected data, and a consensus discussion was held between the researcher and the independent coder to review the findings.

### Ethical considerations

Approval was obtained from the Research Ethics Committee at a university in Johannesburg (reference number REC-01-050-2020) prior to data collection. Permission to conduct the study was also obtained from the relevant stakeholders. All participants in the study received a letter of information and a consent form to consent to participate in the study. Their participation was voluntary, and they were told that they had the right to withdraw from the study at any time without incurring any penalty. Precautionary measures were taken to safeguard the participants with regard to anonymity and confidentiality. The participants were assigned a code to identify them, and their names were not mentioned or recorded during discussions. They were advised of the confidentiality and anonymity of the discussion and responses.

## Results

Four themes emerged during the data analysis. Participants shared positive and negative experiences of their involvement in the fieldwork, as well as their realisation of the value of collaboration between various sectors. They also expressed their wish to do more. The themes are further illustrated in Box 2.

BOX 2 Themes that emerged from research data.

**Major themes**

**Table:** 

Theme 1	Positive experiences related to fieldwork
Theme 2	Negative experiences related to fieldwork
Theme 3	The value of intersectoral collaboration
Theme 4	Desire and capacity to do more

### Theme 1: Positive experiences related to fieldwork

The participants reflected on their positive experiences as a result of being exposed to situations in the different communities included in this research. One participant shared that he found the change of environment from what he is used to especially humbling. The following direct quote supports the experience:

‘For me, what was humbling, it was a cultural shock, it was a different environment that I’m not used to.’ (Participant 1, social worker, male)

Even though the experience of being humbled is seldom positive, this participant emphasised that his perception of the world has broadened, making him aware of other people’s experiences. Not knowing about the existence of environments that differ from his own narrowed his world. Being part of the CoP research thus extended his knowledge and perception base.

Another participant mentioned the experience was remarkable as she discovered how effective collaboration could be during her involvement in this research; the usefulness of working with professional nurses in the community was evident. The following quote is indicative of her experience:

‘Awesome, it was one of those experiences I would not trade for anything in my life and career.’ (Participant 2, social worker, female)

Other participants shared experiences of finding the fieldwork insightful and informative and being grateful for the exposure. The following direct quotes reflect these experiences:

‘It was eye-opening because I have always heard people talking about Alex and also watching about it on TV [*television*], so seeing it for myself was something else.’ (Participant 3, professional nurse, female)

‘I found the field work to be informative because I learnt a lot from the experience of being involved with the different communities.’ (Participant 4, social worker, female)

‘I was appreciative because when I was working with the kids, I realised that they have dreams. They want to become pilots, they want to become firefighters. They stand at the corners, waiting for something to happen … They have goals.’ (Participant 5, professional nurse, female)

The quotes indicate that both social workers and professional nurses gained awareness of how poverty-stricken communities live by being part of the CoP research team. Their community involvement during this project helped them gain their own first-hand experiences, rather than being aware of such circumstances through others’ experiences.

Participants appreciated that some parents were taking good care of their children and that the children revealed resilience in the challenging environments in which they were living. These direct quotes indicate this appreciation:

‘But one thing that was nice and positive was that the presence of the parents, they were taking well care of their children. Their children’s vaccinations were up to date.’ (Participant 6, professional nurse, female)

‘I appreciated that the children are resilient which ever circumstances, in Alex, is the worst, I have never seen anything like this.’ (Participant 5, professional nurse, female)

The following quotes illustrate the warm feeling participants experienced as they reflected on the fieldwork. Participants representing both the social work and nursing professions shared experiences that reflected their career satisfaction:

‘I got fulfilled that they trusted me as a social worker. A few that I referred, they said that “yes, I will take this opportunity to talk.” They realised that it is possible to speak to someone. She was amazed that there is a service.’ (Participant 2, social worker, female)

‘For me, working with kids, how am I going to interact with them? Kids are willing, you explain what you will do, but they respond, even though they are quiet and not talking a lot. They listen, and for them it was a process full of fun. The parents have their own fears. The children have hope.’ (Participant 5, professional nurse, female)

The participants’ feedback confirms their positive experiences because of being exposed to situations in the different communities included in this research.

### Theme 2: Negative experiences related to fieldwork

The participants reported some difficulties while being involved with the fieldwork. Their negative experiences focused on aspects to which they were exposed, and participants revealed how this exposure made them feel. The participants verbalised finding some of the moments difficult, emotionally draining and overwhelming because of the challenges they observed within the communities.

One participant shared:

‘There were moments that were difficult. I don’t feel like a social worker. I was busy with her. At work they phone me, but the participant wanted to keep on talk to me. I realised that this one needs at least six sessions to get into therapy. I didn’t do justice.’ (Participant 7, social worker, female)

This participant referred to a situation where her client was in need to talk and share more about her life challenges; however, the participant was unable to spend more time with the client as she had other commitments.

Another participant mentioned she felt emotionally drained:

‘It became emotionally draining to see and hear the challenges that the parents and children were going through.’ (Participant 9, professional nurse, female)

One participant explained that she found a particular family situation overwhelming, as supported by the following excerpt:

‘Nine people in a shack, granny about 80, 4–5 years old children, granny looked after the children and had to cook for herself. I felt it was too much for the granny and having to look after the children … taxing, especially the granny, COVID. I would probe her, and she said that she is used to the situation. I tried to find more help for her. Did a referral to Bara, she is mostly all by herself. I asked where are the mothers and she said they will sleep and go.’ (Participant 8, social worker, female)

Some of the negative experiences were related to seeing the challenging environment in which the communities lived. This perception was supported by the following direct quotes:

‘I also had a situation that is almost the same. The mother was abusing substances, the granny is looking after the children. This is so emotional, you wish you can do more, but your hands are tied. It is emotional. We are dealing with the cases, they are all the same, you know it is hurting, you must be there for the person.’ (Participant 6, professional nurse, female)

‘I had two fathers who brought the children. It is a young couple. For me what was hurting was the father going through his own emotions, he was intoxicated … he started breaking down, “I am in debts, I’m here for the children. The only thing I can do, is to drink”. I felt so helpless, as he said “I don’t think there is anything you can do for me. I didn’t know what more to do”. You can see the child looking up to the father. It was nine in the morning. He was already drunk. I just thought, the child is a boy, you can see he is looking at the dad, seeing his hero. He said “I don’t think my child knows me being sober. There is a lot happening”. The issue about debt, is like opening a can of worm. He has loan sharks after him. Even if he can go for therapy, he has been through all interventions, the only thing that he still can do is doing something for his children.’ (Participant 2, social worker, female)

Individuals who choose a career that involves human beings have an inherent feeling of responsibility to help individuals, families and communities. When they are unable to practise their profession by serving community members, they become emotionally drained and develop a moral injury, as evident from the above quotes.

### Theme 3: The value of intersectoral collaboration

The professional nurses were accustomed to their normal day-to-day work of seeing patients when they came to the clinics and hospitals. However, they were not always aware of the function of social workers and expressed their appreciation for the role social workers play in the community. The following quotes reflect the professional nurses’ experiences:

‘Because they were seated, I overheard a conversation, because I don’t know how social workers do it. Sometimes the questions you don’t internalise. She [*social worker*] asked if the children go to bed hungry, and the father said, “yes they do”. I developed a different kind of respect for the social workers.’ (Participant 9, professional nurse, female)

‘Yes, I developed a lot of respect for social workers. I am relieved to know what they do in the community, and having someone to refer the children to.’ (Participant 11, professional nurse, female)

One participant alluded to how relieved she was that a social worker was nearby and that she would be able to refer the child’s father. Dealing with the father’s emotions was hard for her, and she was aware social workers are better equipped to deal with parents’ despair:

‘I saw that the father will cry, and I thought, “don’t cry, don’t cry”. I know the social workers are there, and I referred them as I know she was there.’ (Participant 3, professional nurse, female)

Likewise, the social workers expressed their insight into the significance of professional nurses’ work, and the fact that they could work together as a team, enhancing the lives of children and their families within the communities. The following quote supports this theme:

‘[…*S*]ome of the parents asked: “were you at the nurse?” … and someone asked: “oh, did you work together?”’ (Participant 2, social worker, female)

The community was pleasantly surprised that the nurses and social workers worked together; it appeared they appreciated the collaboration.

### Theme 4: Desire and capacity to do more

The participants revealed they wished they could offer more to the communities. They expressed the desire to go beyond the role of collecting data for this study to offer services as the need arose among the different people they met.

This theme was encapsulated in the following direct quotes:

‘To get there with a proper resource list to refer. To have a follow up system and giving them our time and not to refer and give them our time at our office, or at home visits.’ (Participant 2, social worker, female)

‘If we as nurses can do our health education about the vaccination.’ (Participant 6, professional nurse, female)

‘What about us getting a cooler box to do catch-up vaccine.’ (Participant 9, professional nurse, female)

The participants wished they had resources at their disposal to assist the communities with their challenges.

Participants’ feedback confirms that they experienced positive and negative responses related to their fieldwork; they valued intersectoral collaboration and expressed a desire and capacity to do more for the community.

## Discussion

The four themes identified offer insight into the experiences of professional nurses and social workers collaboratively conducting psychosocial and health screenings of children and their families. The study’s findings established that participants experienced both positive and negative responses during the fieldwork.

Participants recognised the value of collaboration between the health and welfare sector and that the health of children and their families can be improved through a collaborative effort. Their disappointment at not being able to do more than just screening children and their families’ psychosocial and health aspects was unmistakable. Generally, both the professional nurses and social workers experienced a sense of empowerment from working together to enhance children and their families’ immediate and long-term health. The findings support the ISHF, WHO, UNICEF and the World Bank’s proposal that multisectoral collaboration can lead to the creation of nurturing environments that integrate various interventions across social sectors to promote better outcomes for children in the early years. Investing in children’s nutrition and health generates short- and long-term benefits and ensures the fulfilment of children’s needs and rights.^8,9^

However, instead of achieving these potential benefits, the community-based health services in primary health care contexts are experiencing challenges in implementing policies as planned by the various stakeholders.^32^ Moosa et al.^33^ concluded that positive sentiments towards the intent of primary health clinics’ outreach teams do exist, but there are concerns about the programme’s management. This study suggests the formalisation and training of community health workers (CHWs). Ramukumba^34^ states that CHWs found meaning in their work because of the positive community response and the good interactions they experienced.

The major obstacle participants mentioned was the CHW programme’s structure and apparent lack of support from the government. There seems to be a paucity of available literature confirming professional nurses’ experiences in conducting home visits, thereby supporting the statements from the professional nurse participants that they were surprised by the circumstances they discovered while working within the research context. Research by Calitz, Roux and Strydom^35^ found that more than half of the social workers in their study felt valued and appreciated, while nearly 27% felt appreciated less than 40% of the time. Interestingly, participants from the welfare sector in the present study made positive statements and reported that it was good to be appreciated by the community and that they felt fulfilled by their experiences.

In addition to participants reporting positive responses from being involved in the fieldwork, some participants shared negative experiences. Nursing is deemed a profession of high moral standards, based on a commitment to supporting others’ well-being.^36^ In South Africa’s public health sector, nurses make up 56% of all healthcare providers, with different models estimating both current and forthcoming nurse shortages. Nurses are critical in addressing the complex burden of disease, the primary health care approach and improving health system performance. However, South Africa does not have consensus regarding national staffing norms. Nursing curricula have also changed over time, and implementing improved health systems has been a drawn-out process, resulting in a lack of responsiveness to communities’ health system needs.^37^ The fact that participants shared feelings of being emotionally drained and unable to help community members illustrates their desire to do more but being unable to do so because of organisational and policy restraints. Consideration and an inclination to help are fundamental in the nursing profession; they allow nurses to develop therapeutic rapport with their patients and deliver nursing care of high value.^38^

Social workers find it difficult to uphold their professional standards as a unified profession because they rely on the state to fund social worker salaries. This situation limits social workers’ capacity to practise professional values free from the state’s agenda. The administrative context in which social workers are employed produces uneven positions for social workers to affirm their professional values through policy support.^39^ Calitz et al.^35^ confirm that South Africa experienced a radical shortage of social workers, which reduced the visible means of support these professionals offer in the community with regard to children and families. Moreover, a study by Azzi-Lessing and Schmidt^40^ maintains that social workers’ home-visiting programmes are still in the development phase. This situation resonates with the participant stating that being exposed to the poverty-stricken community was a cultural shock, as it is an environment unlike his normal working environment. Social workers experience severe negative responses from working in communities where poverty is rife, as illustrated in the stories shared by the participants. Turner^41^ confirms that social workers feel ineffective in the face of poverty affecting children and their families. However, for this article, the collaborative experiences of both the nurses and social workers were explored and the article was written from the viewpoint of nurses.

The number of health professionals in South Africa is insufficient to attend to the 84% of patients who depend on the public sector.^42^ The negative experiences shared by participants are thus understandable, and literature supports their feelings of distress. For this reason, it is vital that health and care professionals like nurses and social workers learn to work together as a team. This notion is referred to as interprofessional collaboration.^42^

Besides the positive and negative experiences, both professional nurses and social workers realised the importance of collaborating and developing trust and mutual respect for their respective professions, evident from the participant quotes. According to Maree and Van Wyk,^43^ in order to improve collaborative practice in healthcare, interprofessional education in health has been recognised as a potential solution. A shift from training various professionals in isolation has been identified as a means to address the challenges in the healthcare system. However, the execution of interprofessional health education in the health disciplines for undergraduate students – to promote teamwork among professionals and improve quality health outcomes – is intricate and difficult to implement. The WHO^44^ recommends interprofessional education while contributing to the depth of education in each profession, learning from each other, with each other and about each other. Learning areas should thus be aligned with generic aspects of healthcare and collaborative practice.

Even though participants expressed appreciation and respect for each other’s professions, they conveyed a desire to do more for the communities than they were able to during the psychosocial and health screenings. Other researchers^45,46^ agree that professional nurses are dissatisfied with their working conditions because of organisational and political influences preventing them from doing more for their communities. Literature also affirms that social workers are vital to communities as they provide their services with honour and commitment and assist the community to the best of their abilities. The social work profession has embraced empowering and promoting the well-being of children and their families.^47^ Van Breda and Addinall^48^ also reported that social workers expressed a need to do more in communities within a developmental social welfare approach, similar to the findings of this research.

### Strength and limitations

The strength of this study relates to the methodology used, as focus group interviews and field notes were employed to collect data. The use of purposive sampling enabled the researcher to select information-rich participants and allowed the latter to share their experiences related to the research topic. A limitation for this study was that a small number of participants were interviewed; however, purposive sampling was used to provide a deep understanding of the participants’ experiences, thereby potentially enhancing the transferability of the findings to related healthcare practitioners. Another limitation was that only professional nurses and social worker experiences were explored and not those of professionals from the education sectors serving children and families for the purpose of improved child well-being. The intention of this article was to share the experiences of representatives from the health -and welfare sectors from nurses’ perspective.

### Recommendations

Both professions want to make life better for the community and expressed satisfaction with and acknowledgement of each profession’s characteristics. However, they are saddened by the circumstances in which communities live, knowing that it will negatively influence the children’s current and long-term health and development. Seemingly, the only condition for health professionals to be successfully involved in interprofessional education is to have a shared goal of improving health outcomes for children and their families.

Based on the findings from this study, the researcher recommends that active collaboration between professional nurses and social workers be initiated, encouraged and supported, not only in undergraduate and postgraduate training but also on the South African policy level. Participants representing both professions reiterated that they were not aware of the role that the other professionals played in the community. Therefore, it is important to motivate greater rapport among the professional nurses and social workers working in the communities, about their functions and responsibilities through platforms like the CoP. Both professions must be encouraged to cross-refer to each other; for example, social workers who identify children who are behind with their vaccinations should refer such families to the nearest community clinics. In cases where professional nurses identify a lack of food security in families, they should refer such children to the social workers. Both professions can also be involved in community development projects to encourage, for example, establishing vegetable gardens for families to become self-sustaining and ensure food security.

It is also recommended that the roles and responsibilities of school social workers be clearly defined by their employers, the larger education system, the DoBE, school governing bodies and school principals, as well as for the social workers themselves working in school health. Conversely, the DoH should ensure practical operationalisation of the ISHP by employing enough relevant professional nurses to work in the community. The ISHP should include collaborative guidelines for both social workers and professional nurses and not just nurses.

## Conclusions

The study’s findings highlighted some positive and negative experiences related to participants’ involvement in the fieldwork. In this article, the collaboration between professional nurses and social workers was reflected on from a nursing perspective, and the importance of disciplined collaboration has been emphasised. Consensus was reached that collaboration between the education, health and welfare sectors is vital to support and promote families’ and children’s health, as evident in the themes. This pilot study confirmed that during COVID-19, many parents who live in deprived circumstances were unable to support their children emotionally and physically. The parents did not have the insight, knowledge and skills to support their children with ongoing education at home during the lockdown. The importance of these health professionals being engaged as a team was therefore highlighted to support families and children.
